# Development and validation of the Chinese family environment influencing physical activity habits scale

**DOI:** 10.3389/fpsyg.2023.1243658

**Published:** 2024-01-16

**Authors:** Xulin Zhang, Jingfei Yan, Weiqiang Zhu, Xiaoya Fu

**Affiliations:** ^1^College of Physical Education and Health, East China Normal University, Shanghai, China; ^2^Ministry of Physical Education, Shanghai Institute of Technology, Shanghai, China; ^3^College of Physical Education, Ningxia Normal University, Guyuan, China

**Keywords:** Chinese adolescents, physical activity habit, family environment, scale development, factor analysis

## Abstract

**Introduction:**

Adolescence represents a pivotal stage in the development of healthy behaviors, where establishing positive physical activity habits can have enduring effects on an individual's overall wellbeing. The ecological model highlights the influence of environmental factors on human behavior, with the family environment playing a significant role in shaping the physical activity habits of adolescents.

**Methods:**

The aim of this scale is to develop a reliable and effective tool, customized for the unique societal context of China, to assess how family factors influence the physical activity habits of Chinese adolescents. Participants were recruited through stratified cluster sampling from 24 secondary schools in six Chinese provinces between October and November 2021, resulting in 1,061 participants. Analysis was conducted on 1,004 valid questionnaires, divided into two samples. Sample 1, consisting of 502 students (248 males and 254 females; M_age_ = 15.5), underwent item analysis and exploratory factor analysis. Sample 2, comprising 502 students (267 males and 235 females; M_age_ = 16.5), underwent confirmatory factor analysis and internal consistency reliability analysis.

**Results:**

Through exploratory factor analysis, we extracted three factors comprising 15 items: “Family Environment Construction” (4 items), “Family Action Support” (6 items), and “Family Health Awareness” (5 items). The Cronbach's alpha values for these factors ranged from 0.890 to 0.894. Confirmatory factor analysis confirmed a satisfactory model fit (CMIN/DF = 1.45, SRMR = 0.027, GFI = 0.991, TLI = 0.989, RMSEA = 0.03).

**Discussion:**

The scale demonstrated strong internal consistency and test-retest reliability, confirming its effectiveness in empirical research. This study holds significant implications for enhancing the physical activity levels of adolescents, promoting their physical and mental wellbeing, enriching their developmental experiences, and contributing to their overall sense of happiness.

## 1 Introduction

Physical activity is defined as any bodily movement produced by skeletal muscles that requires energy expenditure, encompassing activities such as walking, cycling, sports, and recreational activities (World Health Organization, 2022). Physical activity contributes significantly to enhancing overall health and reducing the risk of chronic diseases, including cardiovascular diseases, cancer and diabetes (World Health Organization, 2022). Regular engagement in physical activity plays a crucial role in promoting the health and well-being of individuals, particularly during adolescence, which is a critical period for the development of health behaviors (van Sluijs et al., 2021). Establishing positive patterns of physical activity during this stage can have long-lasting effects on individuals' health and wellbeing throughout their lives.

The application of health behavior theories in physical activity has revealed the role of environmental influences, most commonly “barriers,” “facilitating conditions,” or “contextual influences” (Godin, 1994). Bandura (1986)'s social cognitive theory provides valuable insights into the interplay among environmental, personal, and behavioral factors. The varying impact of these factors is contingent upon the specific activity, individual characteristics, and environmental context. Transitioning to the work of Sallis and Hovell (1990), who introduced a social cognitive model for physical activity behavior, emphasizes the pivotal role of environmental attributes within a framework where multiple determinants interact across various levels. Within this model (Sallis and Hovell, 1990) the “ecological” model of health behavior provides a holistic understanding of how diverse determinants interact across various levels in individuals' physical and socio-cultural environments. The family environment, as a primary socializing agent, holds significant influence adolescents' exercise behaviors and habits (Roque Garay, 2018; Korom et al., 2023). Families provide the foundation for learning and adopting health behaviors (Hunt et al., 2015). Extensive research has demonstrated the impact of the family environment on the formation of exercise habits among adolescents (Mota, 1998; Zovko et al., 2021). For example, factor such as family support (Kuo et al., 2007; Álvarez Muñoz and Hernández Prados, 2023; Hu and Cai, 2023), parental role modeling (Adkins et al., 2017; Do et al., 2023), and the availability of resources and opportunities for physical activity within the family context (Álvarez Muñoz and Hernández Prados, 2023) have been found to be crucial in influencing adolescents' exercise behaviors. The family environment encompasses various aspects, including parental attitudes (Cleland et al., 2011; Whooten et al., 2023), parental involvement (Kuo et al., 2007), family socioeconomic status (Cleland et al., 2011), and the home physical activity environment (Kuo et al., 2007; Cleland et al., 2011). Understanding the influence of the family environment on adolescent exercise behavior can lead to the development of effective interventions and programs to promote physical activity and cultivate exercise habits among adolescent. According to recent statistics, only 20% of Chinese youth aged 5–17 engage in 60 min of moderate to vigorous activity daily, which fall far below the recommendations set by the World Health Organization (Liu, 2023). The *Childhood Blue Book: China Children's Development Report (2021)* reveals an alarming increase of 8.7 percentage points in the rates of overweight and obesity among elementary and middle school students from 2010 to 2019 (Zhao and He, 2023). This highlights the urgent need for interventions promoting physical activity in this population.

However, a lack of scientific measurement and evaluation tools hinders a comprehensive understanding of how families influence adolescents' physical activity and exercise habits. Prior family-focused models such as the Family Influence Scale (Taylor et al., 1994) and Family Eating and Activity Habits Questionnaire (Golan et al., 2006) have quantified influences like parenting practices and role modeling on youth dietary and activity behaviors. However, these Western-developed tools lack cultural adaptation for the Chinese context. They often emphasize adolescent autonomy (Chai et al., 2018) rather than filial piety norms (Deng et al., 2022) This indicates a need for localized measurement models to fully capture family environment dynamics shaping Chinese adolescents' physical activity habits. To measure the influence of the family environment on adolescent physical activity behaviors, the development of a reliable and valid measurement tool is crucial. Firstly, current assessment tools often lack a comprehensive focus on family factors. Several existing studies exhibit limitations in exploring a wide array of family factors. For example, some studies employ retrospective surveys (Kim et al., 2007), diminishing relevance in understanding contemporary family environment impacts. Additionally, a substantial number rely solely on self-reports from adolescents (Henry et al., 2014), neglecting to incorporate parent perspectives. The lack of instruments evaluating various real-time family influences underscores the necessity for a culturally adapted scale that comprehensively addresses this gap. Others summarize influencing dimensions too broadly, failing to provide comprehensive content (McMurray et al., 1993; Joosse et al., 2008; Moreno et al., 2011). Secondly, within the Chinese context, the localization of evaluation tools for practical applications is not ideal, limiting their effectiveness in research. Chinese researchers mainly rely on foreign questionnaires or scales with revisions to measure physical activity habit of the local population. These primarily focus on family health awareness factors, such as individual physical fitness, health status (Wang, 2011), the importance attached to health (Lin, 2019), and the benefits of physical activity (Hu, 2017). Other factors include the family's economic and educational environment (Lv et al., 2020), parents' educational philosophy (Weng, 2015), parents' personality, and children's time monitoring and allocation (Chu and Xiao, 2020). However, these measurements often lack adequate localization to the unique Chinese context and norms. Concepts such as academic pressure (Xie et al., 2004), parenting style (Xie et al., 2022), filial piety (Cao and Tan, 2021) differ between Western and Chinese cultures. Directly applying measurement tools developed abroad could fail to capture family factors shaping physical activity habits among Chinese adolescents and potentially lead to erroneous interpretations.

Therefore, the primary aim of this study is to develop and validate a scale for assessing the influence of the family environment on the formation of physical activity habits among Chinese adolescents. This research holds significant implications: Firstly, it contributes to a comprehensive understanding of how families influence adolescents' physical activity habits and enhances effective promotion of physical activity. Secondly, it facilitates the development of assessment tools better suited to China's local contexts by specifically focusing on family factors relevant to Chinese adolescents. Lastly, the study aims to gain experience localizing and developing measures of how families impact physical activity for Chinese adolescents.

## 2 Materials and methods

### 2.1 Original items for scale development

This study reviewed and analyzed peer-reviewed empirical studies published before January 2023 that employed the social-ecological theory framework to examine how family environment influences adolescents' physical activity habits. The inclusion criteria were: (1) study population of adolescents; (2) investigation of the relationship between family environment variables and adolescent physical activity; (3) use of social-ecological theory to guide the research. Based on the literature review, an interview guide was developed to elicit perspectives on family factors affecting adolescents' activity habits.

The original item pool was created through the following steps: First, extensive potential items grounded in the social-ecological theory and literature were generated through group discussion by the research team. Next, a panel of seven experts in adolescent development and health behavior research evaluated the items for relevance and specificity, with items endorsed by at least five experts retained. The resulting preliminary item pool underwent two rounds of qualitative interviews to obtain target population feedback on the relevance, clarity and construct coverage of the items. The first round involved 2 in-depth individual interviews (approx. 35 mins each) and 4 focus group discussions with 4–5 adolescents per group (approx. 30 mins each), with a total of 18 adolescents. The second round included 4 in-depth individual interviews (approx. 25 mins each) and 5 focus groups of 4–7 adolescents (approx. 35 mins each), with a total of 34 adolescents. Based on the interview data, items were added, eliminated and modified. Finally, the research team held group discussions to reach consensus on the final selection of items for the initial pool.

Subsequently, seven experts in relevant research fields were invited to evaluate the scale. The experts reviewed and examined the content validity index (item-level CVI, I-CVI) of the scale, identified issues, and provided specific modification suggestions. Based on the experts' recommendations, the item content of the scale was modified and revised, resulting in the development of a preliminary version of the “Chinese Adolescents' Physical Activity Habit Formation Scale: Family Environmental Influencing Factors.”

### 2.2 Participants

The questionnaire survey method was employed in this study, taking into account the regional division of China's three major economic belts, to represent adolescents across China's diverse geographic and socioeconomic areas. To minimize selection bias, multi-stage cluster sampling was utilized. Schools were randomly selected from each region, then classes within chosen schools were randomly sampled to obtain a nationally representative, unbiased participant group. The multistage cluster sampling was utilized to select 1,061 participants from 24 middle schools in six provinces across eastern, central and western China. The effective response rate was 94.6%. Among the collected questionnaires 1,004 were deemed valid and randomly divided into two equal groups: sample 1 and sample 2, each consisting of 502 participants. Scale item analysis and exploratory factor analysis were conducted on sample 1 (248 boys, 254 girls; M_age_ = 15.5), while confirmatory factor analysis was performed on sample 2 (267 boys, 235 girls; M_age_ = 16.5).

The recruitment process involved a convenience sample of adolescent students from local schools. All participants' parents or legal guardians provided written informed consent, allowing their children to participate in research. The study received ethical approval from the Ethics Committee of East China Normal University, and this approval ensured that written informed consent was obtained from all participating adolescents and their parents or legal guardians (Ethical approval number: HR 476-2020).

### 2.3 Items and scoring method

Based on previous relevant scientific research findings and expert opinions, a scale was developed to assess the family factors influencing the development of physical activity habits among Chinese adolescents. The scale consists of three factors and 28 items. Factor 1: “Family Environment Construction” (FEC) comprises 8 items; Factor 2: “Family Action Support” (FAS) includes 12 items; and Factor 3: “Family Health Awareness” (FHA) consists of 8 items. The specific content of each factor and its corresponding items can be found in Table 1.

**Table 1:** Initial scale composition.

**Factor**	**Variation**	**Item description**	**References**
Family environment construction (FEC)	Family planning settings	1 Parents can discuss physical activity goals with me	Timperio et al., 2013
		2 Parents can make physical activity plans with me	Nakahori et al., 2016
		3 Parents can make physical activity plans that conform to my actual situation	Qiao, 2011
	Family atmosphere building	1 The family is equipped with sports equipment capable of physical activity	
		2 There is room for physical activity at home	
		3 My parents often tell me about physical activities	
		4 My parents often share their experience of physical activity with me	
		5 Parents often do physical activities at home	
Family action support (FAS)	Example of action	1 Parents can master 1–2 sports skills	Dai and Chen, 2019
		2 My parents often provide guidance when I am physically active	Chu and Xiao, 2020
		3 My parents often do physical activities with me	Bauer et al., 2008
		4 Parents never do physical activities at home	Rhodes et al., 2019
	Emotional support	1 My parents often agitate me when I encounter setbacks in physical activities	
		2 My parents often affirm me when I get achievements in physical activities	
		3 My parents often tolerate me when I have bad emotions in physical activities	
		4 When I am doing physical activities, my parents often stand beside me as an audience	
	Economic support	1 My parents can meet my needs for sports equipment	
		2 My parents can meet my need to watch sports games on the spot	
		3 My parents often give me material rewards after I participate in physical activities	
		4 My parents can meet my requirements for participating in sports interest classes	
Family health awareness (FHA)	Parental lifestyle	1 Physical activity is a part of parents' daily life	Tenjin et al., 2020
		2 Parents attach great importance to healthy lifestyle	Du, 2013
		3 Parents attach great importance to the study of health knowledge	Li and Liang, 2020
		4 Parents don't seem to take part in physical activities at ordinary times	Xie et al., 2004
	Parents' health awareness	1 Parents think that participating in physical activities can improve health	
		2 Parents think that physical activity can relieve stress	
		3 Parents think PE is as important as the main course	
		4 Parents believe that physical activity can promote all-round development	

The core evaluation method employed in the scale is the 5-point Likert scale, where respondents indicate their level of agreement or compliance using the following scale: “1” for completely non-compliant, “2” for not very compliant, “3” for average, “4” for compliant, and “5” for very compliant. It is important to note that reverse-scored item is included in the scale, such as the item “Parents do not seem to normally participate in physical activities within the family health awareness factor, as shown in Table 1. Prior to data analysis, the reverse-scored item in the scale is reversed to ensure consistency in scoring.

### 2.4 Statistical analysis

This study conducted statistical analysis of the data using SPSS24.0 and Mplus 8.3. Initially, descriptive analysis was performed, wherein categorical variables were presented in terms of frequency and percentage, while numerical variables were characterized using the mean and standard deviation (Kim et al., 2020). Subsequently, expert validity evaluation was applied to assess the appropriateness of the scale items, following the methodology proposed by Polit and Beck (2006). The discrimination of each measurement item was tested through item analysis (Raykov and Marcoulides, 2011). Furthermore, exploratory factor analysis was utilized to delve into and optimize the scale structure (Costello and Osborne, 2019), The adequacy of the data for factor analysis was scrutinized using the Kaiser-Meyer-Olkin (KMO) parameter and the Bartlett sphericity test (Kleinbaum, 2013). The reliability of the scale was gauged using Cronbach's coefficient α serving as the reliability measurement index (Tavakol and Dennick, 2011). To verify the scale structure, confirmatory factor analysis was conducted (Brown, 2015), assessing the structural validity of the scale using Composite Reliability and Average Variance Extracted to test combined reliability and convergent validity, respectively. Finally, based on sample 2, content validity was reevaluated.

## 3 Results

### 3.1 Expert assessment of the initial scale

In this study, seven experts were invited to inspect and analyze the survey scale that was designed and compiled in advance. The evaluation criteria used by the experts are outlined in Table 2. According to the guidelines, when the number of expert evaluations exceeds six, the I-CVI value should be >0.78 (Fleiss et al., 2013). After statistically summarizing the evaluations provided by the experts, items with I-CVI values lower than 0.78 in the initial scale were deemed inadequate and subsequently removed. The remaining items that met the requirements I-CVI criteria were retained.

**Table 2:** Expert evaluation results of the initial scale.

**Factor**	**Item**	**Expert evaluation results**							**A**	**n-A**	**I-CVI**	**Evaluation**
		**1**	**2**	**3**	**4**	**5**	**6**	**7**				
Family environment construction (FEC)	1 Parents can discuss physical activity goals with me	4	4	4	4	4	4	4	7	0	1.00	Excellent
	2 Parents can make physical activity plans with me	4	4	4	4	4	4	4	7	0	1.00	Excellent
	3 Parents can make physical activity plans that conform to my actual situation	4	4	4	4	4	4	4	7	0	1.00	Excellent
	4 The family is equipped with sports equipment capable of physical activity	4	4	4	3	3	4	4	5	2	0.71	Good
	5 There is room for physical activity at home	4	4	4	4	4	4	4	7	0	1.00	Excellent
Family action support (FAS)	6 My parents often tell me about physical activities	4	4	3	4	3	4	4	5	2	0.71	Good
	7 My parents often share their experience of physical activity with me	4	3	4	4	3	4	4	5	2	0.71	Good
	8 Parents often do physical activities at home	4	4	4	3	4	3	4	5	2	0.71	Good
	9 Parents can master 1–2 sports skills	3	4	3	4	4	4	3	4	3	0.57	Fair
	10 My parents often provide guidance when I am physically active	4	4	4	4	4	4	4	5	2	0.71	Good
	11 My parents often do physical activities with me	4	4	4	4	4	4	4	7	0	1.00	Excellent
	12 Parents never do physical activities at home	4	4	3	4	4	4	3	5	2	0.71	Good
	13 My parents often agitate me when I encounter setbacks in physical activities	4	4	4	4	4	4	4	7	0	1.00	Excellent
	14 My parents often affirm me when I get achievements in physical activities	4	4	4	4	4	4	4	7	0	1.00	Excellent
	15 My parents often tolerate me when I have bad emotions in physical activities	4	4	4	4	4	4	4	7	0	1.00	Excellent
	16 When I am doing physical activities, my parents often stand beside me as an audience	4	3	4	4	4	3	4	5	2	0.71	Good
	17 My parents can meet my needs for sports equipment	4	4	4	4	4	4	4	7	0	1.00	Excellent
	18 My parents can meet my need to watch sports games on the spot	4	3	4	4	4	3	4	5	2	0.71	Good
	19 My parents often give me material rewards after I participate in physical activities	4	4	4	4	4	4	4	7	0	1.00	Excellent
	20 My parents can meet my requirements for participating in sports interest classes	4	4	4	4	4	4	4	5	2	0.71	Good
Family health awareness (FHA)	21 Physical activity is a part of parents' daily life	4	3	4	4	3	4	4	5	2	0.71	Good
	22 Parents attach great importance to healthy lifestyle	4	4	4	4	4	4	4	7	0	1.00	Excellent
	23 Parents attach great importance to the study of health knowledge	4	4	3	4	4	4	3	5	2	0.71	Good
	24 Parents don't seem to take part in physical activities at ordinary times	4	4	3	4	4	4	3	5	2	0.71	Good
	25 Parents think that participating in physical activities can improve health	4	4	4	4	4	4	4	7	0	1.00	Excellent
	26 Parents think that physical activity can relieve stress	4	4	4	4	4	4	4	7	0	1.00	Excellent
	27 Parents think PE is as important as the main course	4	4	4	4	4	4	4	7	0	1.00	Excellent
	28 Parents believe that physical activity can promote all-round development	4	4	4	4	4	4	4	7	0	1.00	Excellent

Based on expert feedback, inappropriate items were removed. The qualified and reasonable scale items were compiled into a test scale, resulting in a preliminary measurement scale that demonstrated expert validity. The preliminary measurement scale was then evaluated by experts, and items such as items 4, 6, 7, 8, 9, 10, 12, 16, 18, 20, 21, 23, and 24, which had low I-CVI values 0.78 or below, were excluded. The remaining 15 items were retained for further analysis.

### 3.2 Item analysis

After the evaluation by experts, the original 28 items of the scale were reduced to 15 items. In sample 1, which consisted of the 502 valid adolescent questionnaires, the average score for each adolescent's family environment factor was calculated based on the scoring method using the 15-items pre-test scale. The total scores of all subjects were ranked in ascending order. The low group was defined as the material information below 27%, the middle group as the data between 27% and 73%, and the high group as the data above 73% (Kelley, 1939).

Independent samples *t-*test were conducted to determine if there were significant differences between the high and low groups. Table 3 presents the analysis results of the family environment factor subscale items. The family environment factor subscale was analyzed using the critical ratio analysis method based on various indicators. The score for the first 27% was 147, while the score for the last 27% is 139. After conducting the resolution coefficient test and *t-*test, items with *t*-values > 3 and *P* < 0.001 were retained. As a result, item Q29 was eliminated, and the scale was reduced to 15 items.

**Table 3:** Inspection results of scale items.

**Item**	**Extreme group comparison**	**Item-total correlation test**			**Homogeneity test**		**Substandard index**	**Note**
	**Critical value**	**ITC**	**CITC**	**CAID**	**Commonalities**	**Factor loading**		
Q1	20.876	0.709^**^	0.650	0.901	0.827	0.859	0	Retain
Q2	20.794	0.700^**^	0.642	0.902	0.802	0.844	0	Retain
Q3	20.589	0.709^**^	0.652	0.901	0.765	0.810	0	Retain
Q4	18.125	0.641^**^	0.570	0.904	0.652	0.759	0	Retain
Q5	19.139	0.659^**^	0.595	0.903	0.515	0.634	0	Retain
Q6	19.081	0.689^**^	0.629	0.902	0.696	0.800	0	Retain
Q7	19.756	0.735^**^	0.685	0.900	0.806	0.858	0	Retain
Q8	19.165	0.694^**^	0.641	0.902	0.707	0.801	0	Retain
Q9	19.688	0.722^**^	0.672	0.901	0.677	0.755	0	Retain
Q10	**0.627**	**0.034**	**−0.001**	**0.914**	0.985	0.992	4	**Delete**
Q11	14.803	0.606^**^	0.535	0.905	0.554	0.712	0	Retain
Q12	15.818	0.655^**^	0.593	0.903	0.655	0.760	0	Retain
Q13	16.800	0.646^**^	0.586	0.903	0.772	0.854	0	Retain
Q14	18.175	0.687^**^	0.637	0.902	0.770	0.834	0	Retain
Q15	17.022	0.681^**^	0.622	0.902	0.716	0.795	0	Retain
Q16	14.539	0.579^**^	0.514	0.906	0.629	0.770	0	Retain
Judgment criteria	≥3.0	≥0.40	≥0.40	≤ 0.92	≥0.20	≥0.45		

^**^p < 0.01.

### 3.3 Exploratory factor analysis and optimization of the scale

The study first tested the suitability of the remaining 15 items related to family environment factors for factor analysis. The Kaiser-Meyer-Olkin (KMO) value was found to be 0.916, which is >0.8, indicating that the variables are suitable for factor analysis. The Bartlett's sphericity test yielded an approximate chi-square value of 4,704.563 with a significance of 0.000, meeting the significance level (*P* < 0.05) (Table 4). These results suggest that the items are not independent and that there are common factors present in the overall correlation matrix, supporting the suitability of factor analysis for the “Chinese Adolescents' Physical Activity Habit Formation Scale: Family Environmental Influencing Factors.”

**Table 4:** Kaiser-meyer-olkin test and bartlett sphericity test.

**Kaiser-meyer-olkin (KMO)**	**Bartlett sphericity test**		
	**Approx. Chi-Square**	**df**	**Sig**.
0.916	4,704.563	105	0.000

Principal component analysis was conducted to extract common factors with eigenvalues >1, and items with factor loadings exceeding 0.5 were retained. The exploratory factor analysis results in Table 5 indicate that each item's communalities are higher than 0.4, indicating effective extraction of research item information. Through this process, a total of three factors were obtained, each with eigenvalues >1. The variance explanation by these three factors after rotation was 25.938%, 23.747%, and 20.410%, respectively, with a cumulative variance explanation of 25.938%, 49.686% %, and 70.096%. These factors effectively capture the relevant information. The optimized scale consists of 15 items, which were categorized into three factors.

**Table 5:** Results of exploratory factor analysis.

**Item**	**Component**			**Communalities**
	**1**	**2**	**3**	
Q1 Parents can discuss physical activity goals with me			0.860	0.827
Q2 Parents can make physical activity plans with me			0.846	0.802
Q3 Parents can make physical activity plans that conform to my actual situation			0.810	0.764
Q4 There is room for physical activity at home			0.759	0.652
Q5 My parents often do physical activities with me	0.635			0.512
Q6 My parents often agitate me when I encounter setbacks in physical activities	0.799			0.695
Q7 My parents often affirm me when I get achievements in physical activities	0.856			0.800
Q8 My parents often tolerate me when I have bad emotions in physical activities	0.799			0.701
Q9 My parents can meet my needs for sports equipment	0.755			0.676
Q10 My parents can meet my need to watch sports games on the spot	0.713			0.546
Q11 Parents attach great importance to healthy lifestyle		0.759		0.655
Q12 Parents think that participating in physical activities can improve health		0.854		0.771
Q13 Parents think that physical activity can relieve stress		0.834		0.770
Q14 Parents think PE is as important as the main course		0.795		0.715
Q15 Parents believe that physical activity can promote all-round development		0.769		0.628
Eigen value (Rotated)	3.891	3.562	3.062	
% of Variance (Rotated)	25.938	23.747	20.410	
Cumulative % of Variance (Rotated)	25.938	49.686	70.096	

Factor 1 comprises 6 items (Q1, Q2, Q3, Q4, Q5, and Q6) that reflect family members' support, encouragement and rewards, and it is named “Family Environment Construction” (FEC). Factor 2 consists of 5 items (Q7, Q8, Q9, Q10, and Q11) that primarily reflect the parents' healthy lifestyle and their recognition of healthy behaviors, leading to its naming as “Family Action Support” (FAS). Factor 3 includes 4 items (Q12, Q13, Q14, and Q15) that reflect family-related information on physical activity, goal planning, and the creation of physical activity space, thus termed “Family Health Awareness” (FHA).

### 3.4 Reliability analysis of scale content

As shown in Table 6, presents the results of the internal consistency analysis of the measured data. The Cronbach α values for the three dimensions “FEC,” “FAS,” and “FHA” are 0.894, 0.890, and 0.894, respectively. The Cronbach α value of the overall scale is 0.914. These values indicate high reliability, as each dimension and the total scale surpass the recommended threshold of 0.8. Furthermore, the overall scale demonstrates excellent reliability with a value exceeding 0.9. These findings indicates that the “Chinese Adolescents' Physical Activity Habit Formation Scale: Family Environmental Influencing Factors” is reliable and effectively captures the family environment factors that influence the development of physical activity habits among adolescents.

**Table 6:** Reliability analysis results of the scale based on sample 1.

**Factor**	**Cronbach α**	**Item**
Family environment construction	0.894	4
Family action support	0.890	6
Family health awareness	0.894	5
Family environmental factors	0.914	15

Primarily, with the application of Cronbach α, the corrected item-total correlation (Corrected Item-Total Correlation, CITC) and the Cronbach's α coefficient after item deletion (Cronbach's Alpha if Item Deleted, CAID) to determine the structure and dimensions of the research. The reliability was quantified, and Table 7 indicates the results of the reliability analysis based on sample 2. It shows that the CITC value of each measurement item is >0.4, the CAID value is lower than the Cronbach's α coefficient of the corresponding variable, the Cronbach's α of FEC, FAS, and FHA is higher than 0.7, and the total Cronbach's α coefficient of the scale is 0.915. The analysis results indicate that the family environment factor and its dimensions have high reliability. This demonstrates that the actual situations of the selected samples are relatively stable and consistent.

**Table 7:** Reliability analysis results of the scale based on sample 2.

**Reliability**					
**Factor**	**Item**	**CITC**	**SMC**	**CAID**	**Cronbach** α
Family environment construction (FEC)	FEC1	0.756	0.573	0.822	0.871
	FEC2	0.734	0.553	0.830	
	FEC3	0.748	0.569	0.825	
	FEC4	0.661	0.440	0.861	
Family action support (FAS)	FAS1	0.641	0.418	0.876	0.886
	FAS2	0.704	0.527	0.866	
	FAS3	0.752	0.604	0.858	
	FAS4	0.742	0.578	0.860	
	FAS5	0.746	0.561	0.860	
	FAS6	0.625	0.415	0.879	
Family health awareness (FHA)	FHA1	0.657	0.433	0.868	0.880
	FHA2	0.770	0.616	0.841	
	FHA3	0.781	0.620	0.840	
	FHA4	0.723	0.541	0.852	
	FHA5	0.645	0.423	0.870	

### 3.5 Confirmatory factor analysis of the scale

The validity of the research structure and its dimensions was assessed through Confirmatory Factor Analysis (CFA) (Wu, 2010). The results of confirmatory factor analysis, as shown in Table 8, indicate a good fit with χ^2^ = 126.175, df = 87, χ^2^/df = 1.45, RMSEA = 0.03, SRMR = 0.027, CFI = 0.991, TLI = 0.989. These indices meet the fitting requirements established by Hu and Bentler (1999). The χ^2^/df value falls within an acceptable range below 5 (Bentler and Bonett, 1987). In this study, suggesting that the first-order 3-factor confirmatory factor analysis model adequately represents the home environment based on the data. Therefore, the confirmatory factor model demonstrates a sound structure.

**Table 8:** Results of confirmatory factor analysis.

**Factor**	**Item**	**CFA**		
		**Standard**	**CR**	**AVE**
Family environment construction (FEC)	FEC1	0.833	0.872	0.631
	FEC2	0.806		
	FEC3	0.820		
	FEC4	0.714		
Family action support (FAS)	FAS1	0.684	0.889	0.572
	FAS2	0.758		
	FAS3	0.819		
	FAS4	0.806		
	FAS5	0.790		
	FAS6	0.667		
Family health awareness (FHA)	FHA1	0.704	0.883	0.603
	FHA2	0.838		
	FHA3	0.849		
	FHA4	0.792		
	FHA5	0.686		

The standardized factor loading of the items ranged from 0.667 to 0.849, and the composite reliability (CR) value for each variable exceed 0.7. These findings indicate that all variables analyzed exhibited desirable composite reliability. Additionally, the average variance extracted (AVE) of each variable exceeds 0.5, indicating good convergent validity. In summary, the family environment factor and its dimensions demonstrated good reliability and validity in this study.

### 3.6 Test-retest reliability

In this study, we surveyed a sample of 56 high school students using the Chinese Youth Activity Habit Formation Scale. To prevent the results of the initial test from affecting the second test, we introduced a 2-week interval between the pre-test and post-test. The retest reliability coefficient ranged from 0.79 to 0.84, and the correlation coefficients for the three dimensions were 0.832, 0.863, and 0.832, respectively. All these coefficients surpassed 0.7, with an overall coefficient of 0.890. The retest correlation coefficients of the scale meet the necessary criteria, confirming that the reliable retest reliability of Chinese Adolescents' Physical Activity Habit Formation Scale: Family Environmental Influencing Factors is reliable and fulfills the retest requirements.

## 4 Discussion

### 4.1 Results interpretation

Existing studies have predominantly concentrated on family factors influencing adolescents' physical activity. For instance, research have delved into the impact of parental behaviors, such as role modeling (Madsen et al., 2009; Yao and Rhodes, 2015) and parental support (Edwardson et al., 2013; Langer et al., 2014) on youth activity levels. Other have investigated the effects of the family's physical home environment on adolescent behaviors (Trost et al., 2003; Spurrier et al., 2008). However, there has been limited theoretical discussion and few studies exploring factors within the family environment that contribute to the development of adolescents' physical activity habits.

The findings of this study make several notable contributions by identifying three key family environment factors influencing Chinese adolescents' physical activity habits: Family Environment Construction (FEC), Family Action Support (FAS), and Family Health Awareness (FHA). The identification of these specific elements provides new evidence that aspects of the family environment play a crucial role in shaping activity patterns during adolescence. Family Environment Construction (FEC) appears to be a particularly relevant factor in China, possibly due to the cultural emphasis on parental management of children's time. Furthermore, Family Environment Construction (FEC) was found to impact activity time, type, space and interests, aligning with previous literature that illustrates the multifaceted influences of the family environment on youth behaviors (McIver et al., 2009). Family Action Support (FAS) positively predicted physical activity, aligning with previous research that indicates family support enhances adolescent self-efficacy, facilitating participation (Quarmby and Dagkas, 2010; Dai and Chen, 2019). This underscores the necessity for interventions aimed at enhancing parental support. Moreover, results from Family Health Awareness (FHA) revealed that parental modeling positively influenced adolescents' activity levels (Ren et al., 2012; Sigmundová et al., 2014), highlighting the necessity for widespread public health messaging regarding parental impacts. Overall, these findings underscore the family environment's role as a key determinant of healthy physical activity habits during adolescence in China, while also suggesting cultural variations in the relative strength of specific family influence factors.

To address the limitations of using foreign-related scales in measuring family environment factors influencing the formation of physical activity habits, this study focuses on addressing issues such as poor measurement relevance and a failure to capture the key elements affecting habit formation. Drawing on classic questionnaires and scales from both domestic and international sources, this study developed a localized “Chinese Adolescents' Physical Activity Habit Formation Scale: Family Environmental Influencing Factors.”

While the scale development process was rigorous, a key innovation lies in the cultural specificity tailored to Chinese adolescents, differentiating it from existing family influence scales. For instance, Western scales often emphasize adolescent autonomy (Chai et al., 2018), while this scale captures Chinese cultural values such as filial piety (Peng and Li, 2023) and parental authority over play time (Deng et al., 2022). The localized conceptualization enhances the validity of measuring family dynamics influencing physical activity behaviors among Chinese youth.

The scale development process was both rigorous and comprehensive. It drew on previous research findings, used the Harman single-factor test to assess common method bias, conducted item analysis to test item discrimination, explored and optimized the scale's structure, and employed Cronbach's α as a reliability measurement index. The scale's structure was further validated through confirmatory factor analysis, and CR and AVE were used to assess the scale's reliability and validity, respectively.

This study systematically refined the initial scale, measuring family environmental influences on physical activity among Chinese adolescents. Expert advice was incorporated to optimize the questionnaire, enhancing its precision. Refinements involved consolidating related factors, revising dimensions, and eliminating items through rigorous statistical testing and expert review. Specifically, factors related to activity space design and atmosphere creation were combined into “space atmosphere creation.” The initial 28 items were reduced after an evaluation process that included content validation by experts and subsequent statistical analysis. Items such as Q4, Q6, Q16, and Q23 were eliminated due to inadequate relevance based on an item-CVI threshold. Q29 was deleted due to poor discrimination between respondent groups. This iterative refinement process ensured that only the most valid and reliable items were retained. Specifically, items with I-CVI scores below 0.78 were eliminated to retain the most pertinent survey questions. The remaining items then underwent additional evaluation of discrimination, reliability, and validity, resulting in the removal of any that did not meet psychometric standards. This rigorous scale development methodology focused on refining the initial family environmental influences scale through expert input and statistical testing. The adjustments successfully optimized item content and dimension structure while eliminating inadequate components. This systematic approach resulted in a 15-item scale demonstrating strong psychometric properties.

This study developed a culturally-adapted scale assessing family environmental influences on Chinese adolescents' physical activity through a rigorous multiphase process involving expert reviews and statistical optimization. The methodology ensured retention of only the most valid and reliable items, resulting in a precise, highly reliable 15-item scale tailored to the Chinese context, as shown in Table 9. This newly developed scale makes important theoretical contributions by filling a significant research gap—the prior lack of quantitative models customized to assess family environment influences specific to Chinese adolescents. Availability of a validated tool tailored to the Chinese context represents an essential advancement, enabling more rigorous investigation of culturally-specific familial factors affecting youth physical activity. Practically, the scale provides an empirical foundation to inform the development of culturally-appropriate family-based interventions aimed at modifying key environment elements to promote healthy adolescent activity habits.

**Table 9:** Formal scale.

**Factor**	**No**.	**Item**
Family environment construction (FEC)	FEC1	Q1 Parents can discuss physical activity goals with me
	FEC2	Q2 Parents can make physical activity plans with me
	FEC3	Q3 Parents can make physical activity plans that conform to my actual situation
	FEC4	Q4 There is room for physical activity at home
Family action support (FAS)	FAS1	Q5 My parents often do physical activities with me
	FAS2	Q6 My parents often agitate me when I encounter setbacks in physical activities
	FAS3	Q7 My parents often affirm me when I get achievements in physical activities
	FAS4	Q8 My parents often tolerate me when I have bad emotions in physical activities
	FAS5	Q9 My parents can meet my needs for sports equipment
	FAS6	Q10 My parents can meet my need to watch sports games on the spot
Family health awareness (FHA)	FHA1	Q11 Parents attach great importance to healthy lifestyle
	FHA2	Q12 Parents think that participating in physical activities can improve health
	FHA3	Q13 Parents think that physical activity can relieve stress
	FHA4	Q14 Parents think PE is as important as the main course
	FHA5	Q15 Parents believe that physical activity can promote all-round development

In conclusion, the rigorous methodology undertaken to develop and optimize this culturally-adapted scale, coupled with its enhanced precision and reliability, represents an important contribution. The scale will allow deeper investigation of Chinese familial determinants shaping youth activity patterns. Additionally, findings generated can inform family-focused intervention strategies to promote healthy adolescent physical activity behaviors in China.

### 4.2 Limitations and areas for future research

In this study, it is crucial to acknowledge certain limitations. Firstly, despite conducting a thorough review and inclusion of item sources during the formation of the item pool, with a concerted effort to encompass a wide range of potential family factors, some items were still excluded based on expert evaluation, potentially resulting in the oversight of certain assessment details. Therefore, it is imperative to recognize the significance of further enhancing the scale's specificity and intricacy in future research, building upon the foundation established in this study.

Secondly, while our comprehensive survey covered multiple cities across diverse regions of China, validating the universality and efficacy of the scale, there is still room for expanding the sample size. Obtaining a more diverse range of samples would contribute to further confirming the scale's applicability.

Thirdly, given the focus of our study on the specific Chinese context, congruent with Chinese policy and cultural background, potential limitations might arise concerning the scale's practicality in other cultural and national contexts.

Notwithstanding these limitations, the scale holds substantial research value in terms of fostering localization and comprehensiveness, aligning seamlessly with the initial objectives. It represents the most extensive scale developed to date for assessing the impact of family environmental factors on the formation of physical activity habits among Chinese adolescents. Consequently, our scale provides invaluable insights into the cultivation of physical activity levels among Chinese adolescents.

## 5 Conclusion

In conclusion, this study developed and validated the “Chinese Adolescents' Physical Activity Habit Formation Scale: Family Environmental Influencing Factors,” comprising three key factors: “Family Environment Construction” (FEC), “Family Action Support” (FAS), and “Family Health Awareness” (FHA). Reliability and validity analysis indicate this scale provides an objective and effective tool to measure family environmental influences on adolescent physical activity habits.

In summary, the development and validation of the “Chinese Adolescents' Physical Activity Habit Formation Scale: Family Environmental Influencing Factors” represents a significant advancement for research on family determinants of physical activity and wellbeing among Chinese youth. The availability of a reliable and valid scale tailored to the Chinese adolescent population will enable a more robust quantitative investigation into how family environmental factors, unique to the Chinese cultural context, shape physical activity habits. This culture-specific tool can inform targeted interventions involving Chinese families, schools, and communities to promote participation in regular physical activity among adolescents, with significant implications for improving youth wellbeing at the national level in China. Overall, the scale provides an essential culturally grounded tool for deepening understanding and improving key family-level determinants of healthy adolescent development in China.

## Data availability statement

The original contributions presented in the study are included in the article/supplementary material, further inquiries can be directed to the corresponding author.

## Ethics statement

This study has been reviewed and approved by the Ethics Committee of East China Normal University (HR 476-2020). The studies were conducted in accordance with local legislation and institutional requirements. Written informed consent for participation in this study was provided by the participants' legal guardians/next of kin.

## Author contributions

XZ, JY, WZ, and XF contributed to the conception and design of the study. JY and XF were involved in implementing the study and data collection. XZ undertook data analysis and wrote the first draft of the manuscript. XZ, JY, and WZ polished and revised the draft. All authors contributed to the article and approved the submitted version.
