# Corrigendum: Development and validation of the Chinese family environment influencing physical activity habits scale

**DOI:** 10.3389/fpsyg.2024.1521605

**Published:** 2024-11-18

**Authors:** Xulin Zhang, Jingfei Yan, Weiqiang Zhu, Xiaoya Fu

**Affiliations:** ^1^College of Physical Education and Health, East China Normal University, Shanghai, China; ^2^Ministry of Physical Education, Shanghai Institute of Technology, Shanghai, China; ^3^College of Physical Education, Ningxia Normal University, Guyuan, China

**Keywords:** Chinese adolescents, physical activity habit, family environment, scale development, factor analysis

In the published article, there was an error in the Funding statement. The statement was previously written as, “This study was funded by the 2023 Youth Fund for Humanities and Social Science Research of the Ministry of Education, research title: “Model Construction and Empirical Research on Influencing Factors of physical Activity Habit Formation of Chinese Adolescents” (Project No. 23XJC890048)”, with the project number incorrectly written as “23XJC890048”. The correct Funding statement appears below.

